# “U-shaped” mesoportal jump graft to manage portal vein thrombosis during liver transplantation: A case report

**DOI:** 10.1016/j.ijscr.2020.04.098

**Published:** 2020-05-15

**Authors:** Damiano Patrono, Sara Salomone, Carla Guarnaccia, Francesco Tandoi, Francesco Lupo, Paolo Fonio, Renato Romagnoli

**Affiliations:** a General Surgery 2U – Liver Transplant Unit, A.O.U. Città della Salute e della Scienza di Torino, University of Torino, Torino, Italy; b Radiology Department, A.O.U. Città della Salute e della Scienza di Torino, University of Torino, Torino, Italy

**Keywords:** PVT, portal vein thrombosis, LT, liver transplantation, SMV, superior mesenteric vein, D1, first duodenum, MELD, model for end-stage liver disease, CT, computed tomography, IVC, inferior vena cava, TIPS, transjugular portosystemic shunt, Portal vein thrombosis, Yerdel classification, Jump graft, Iliac bifurcation, Case report

## Abstract

**Introduction:**

Once considered a contraindication to liver transplantation, portal vein thrombosis still represents a significant challenge to the liver transplant surgeon. Yerdel grade 3 thrombosis is usually managed by interposing a donor iliac vein jump graft between graft portal vein and distal superior mesenteric vein. Venous patch is normally placed in a retrogastric position to avoid its kinking.

**Presentation of case:**

We report a new technical variant of standard mesoportal jump graft, in which a U-shaped graft was obtained using iliac bifurcation. This technique was used to manage a case of grade 3 portal vein thrombosis in which portal vein was unsuitable due to severe pylephlebitis and pylorus dissection had to be abandoned due to inflammatory changes issue of chronic pancreatitis. The venous patch was of sufficient length and shape to bypass pancreatic head and first duodenum, avoiding the need for its retrogastric placement and pylorus dissection.

**Discussion:**

This case is a further demonstration that technical approach to portal vein thrombosis must be tailored according to its extent and surgical scenario. In selected cases, use of a curved U-shaped jump graft may represent a valuable option.

**Conclusion:**

This technical option should be included among options for the management of portal vein thrombosis and be part of the armamentarium of liver transplant surgeon.

## Highlights

• Portal vein thrombosis increases the technical difficulty of liver transplantation.
• Surgical technique should be adapted to the extent of thrombosis, presence of collaterals and of porto-systemic shunts.
• In particular cases, a mesoportal jump graft obtained using iliac bifurcation may represent a valuable technical option.

## 1 Introduction

Non-tumoral portal vein thrombosis (PVT) is observed in 5%–26% of patients candidate to liver transplantation (LT), with 2% of them presenting complex PVT [1]. Portal vein thrombosis has long been considered as a contraindication for LT due to increased technical difficulty and inferior outcomes, but several technical options for its management during LT have been developed, ranging from low dissection of the portal vein +/− thrombectomy to cavoportal hemitransposition [2,3]. Technical approach to PVT mainly depends on PVT extension, availability of large portal vein collaterals and presence of spontaneous or surgical portosystemic shunt, assessed during pre-LT work-up and intraopearatively [4]. Several PVT classifications have been proposed, with the one proposed by Yerdel el al. [5] being one of the most intuitive and widely adopted.

In Yerdel grade 3 PVT, i.e. complete thrombosis of the portal vein andproximal superior mesenteric vein (SMV), one technical option is distal dissection of the SMV and mesoportal anastomosis using an interposition jump graft. To avoid kinking, the graft normally placed through the transverse mesocolon and posterior to the first duodenum (D1) and the pylorus [2,[5], [6], [7]]. In patients with PVT and presumably severe portal hypertension, D1 dissection or Kocher maneuver may be particularly challenging due to the presence of large and fragile varices.

We present here a novel technique of mesoportal jump graft to manage Yerdel 3 PVT during LT. In the case reported herein, a “U-shaped” donor iliac vein jump graft including iliac bifurcation and the proximal segment of iliac veins was used to get over the pancreatic head and D1, avoiding the need for D1 dissection or Kocher maneuver. The case is reported in line with the SCARE criteria [8].

## 2 Presentation of case

**Fig. 1:**
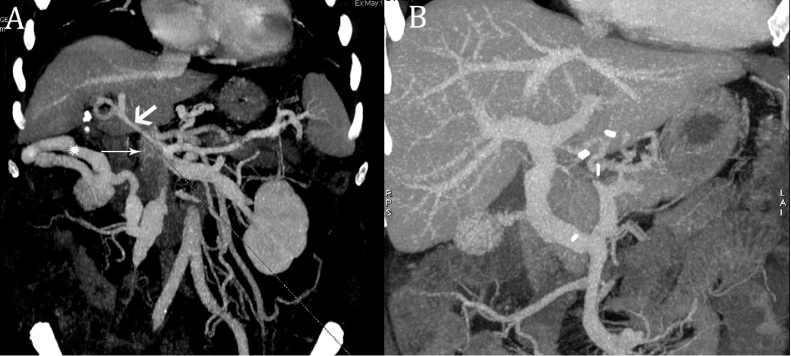
Multiplanar contrast-enhanced computed tomography (CT) 3D reconstructions. A: pretransplant CT showing grade 3 portal vein thrombosis, with proximal involvement of the superior mesenteric vein (thin arrow). A thin portal vein collateral is identified at the hepatoduodenal ligament (thick arrow), as well as a large portosystemic shunt (asterisk) arising from distal superior mesenteric vein and draining into the inferior vena cava, below the outlet of right renal vein. B: post-transplant CT showing patency and position of the mesoportal jump graft.

Patient was a 49-year-old male with end-stage alcohol-related liver disease and a history of several hospital admissions due to decompensated ascites and hepatic encephalopathy. He was referred for LT in May 2019 with creatinine 0.93 mg/dl, total bilirubin 11.2 mg/dl and INR 2.28 and a model of end-stage liver disease (MELD) score of 25. Pre-LT computed tomography (CT) highlighted the presence of a large thrombus in the portal vein and proximal SMV, sparing the splenic vein and the distal SMV (Fig. 1A). CT also showed large a thin portal vein collateral, not suitable for portal vein anastomosis, and a large spontaneous mesocaval shunt draining into the inferior vena cava (IVC) below the outlet of the right renal vein. Treatment with low molecular weight heparin was started (enoxaparin 6000 IU b.i.d.) but it was not tolerated.

Transjugular intrahepatic portosystemic shunt was deemed unfeasible due to the low extension of PVT. Based on PVT extension, LT was deemed feasible by using a mesoportal jump graft interposed between SMV and graft portal vein, the back-up options being renoportal anastomosis or cavoportal hemitransposition. At waitlisting, his MELD score was 29 (creatinine 1.12 mg/dl; total bilirubin 13.0 mg/dl; INR 2.77).

**Fig. 2:**
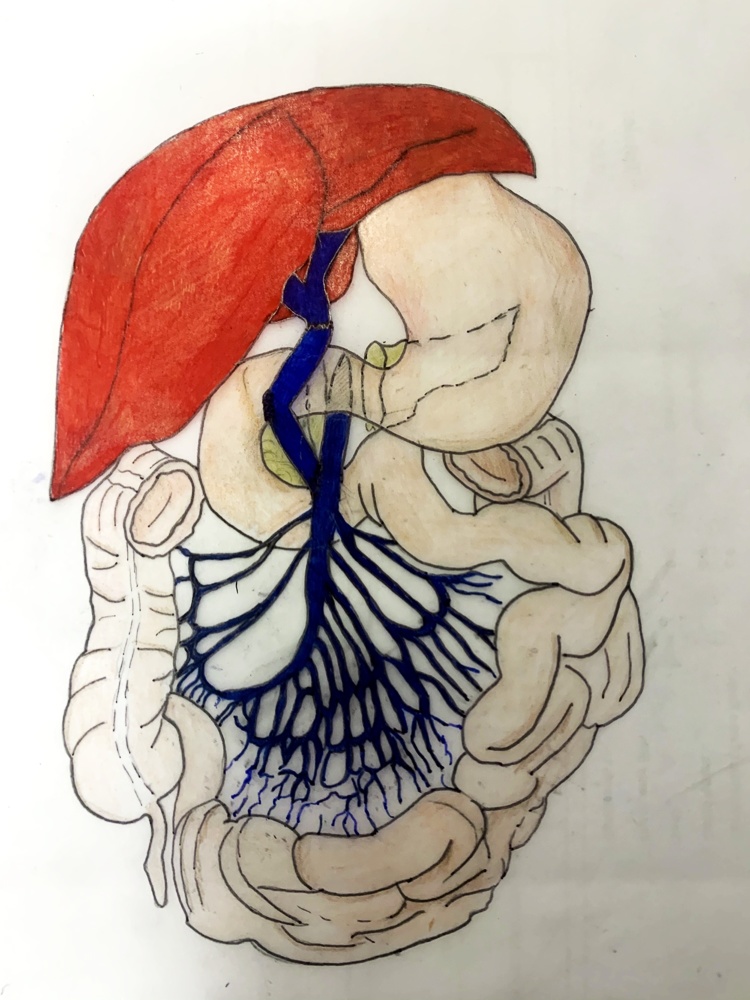
Drawing of the U-shaped mesoportal jump graft.

**Fig. 3:**
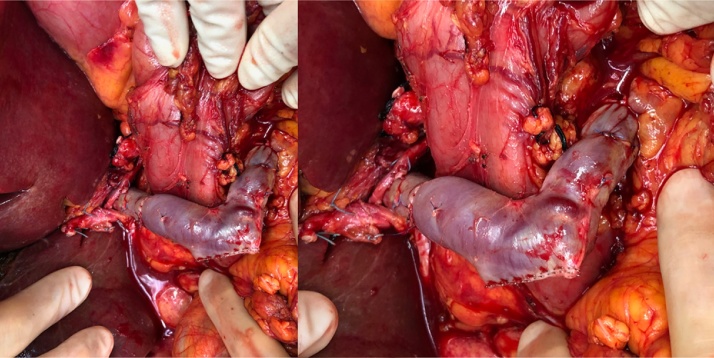
Intraoperative pictures at lower (A) and higher (B) magnification, showing the position of mesoportal jump graft in relation to the pancreatic head and first duodenum.

During organ retrieval, iliac arteries and veins, including aortic and IVC bifurcations, were procured. At liver transplant, long-standing PVT and presence of large portosystemic collaterals were confirmed. As shown by pre-LT imaging, a large mesocaval shunt originated at the level of the gastrocolic trunk of Henle and drained into infrarenal IVC. There was a picture of severe chronic pancreatitis, with peripancreatic inflammatory changes. After dissection of the hepatoduodenal ligament, portal vein appeared to be involved by severe pylephlebitis, with thickened and inflammatory walls and minimal residual lumen. A low dissection of splenomesenteric confluence and an eversion thrombectomy were carried out, which were partially successful in re-establishing a satisfactory flow into the previously obstructed vessel. However, due to the extremely narrowed residual lumen and the thickening of the vessel wall, portal vein was judged unsuitable for anastomosis and abandoned. As originally planned, we decided to perform portal vein anastomosis using an interposition graft between the SMV and graft portal vein. To avoid prolonging cold ischemia time, artery anastomosis was performed first. After graft reperfusion, SMV was dissected distally to the gastrocolic trunk of Henle for approximately 4 cm, preserving the large mesocaval shunt. Preparation of the retrogastric tunnel was abandoned due to the severe inflammatory reaction issue of chronic pancreatitis. A U-shaped donor iliac interposition patch was then prepared by suturing IVC about 2 cm above the iliac bifurcation using a vascular stapler (Endopath ETS-Flex45 mm, Ethicon Endo-surgery, Cincinnati, OH) and by stitching small collaterals originating from common iliac veins. SMV was tangentially clamped and an end-to-side anastomosis between the venous patch and the SMV was performed using a 5/0 polypropylene suture, followed by an end-to-end anastomosis between the venous patch and graft portal vein using the same material (Fig. 2). After clamps removal, the jump graft appeared as optimally bypassing the duodenum and the pancreatic head, in the absence of any tension on the reconstructed vascular axis (Fig. 3). Portal flow assessed by transit time flow measurement (MiraQ Vascular, Medistim®, Oslo, Norway) was 1500 mL/min (107 mL/min/100 g of graft weight). Intraoperative Doppler ultrasound examination confirmed regular graft perfusion. Finally, biliary reconstruction by an end-to-end hepatico-choledocostomy was performed.

**Fig. 4:**
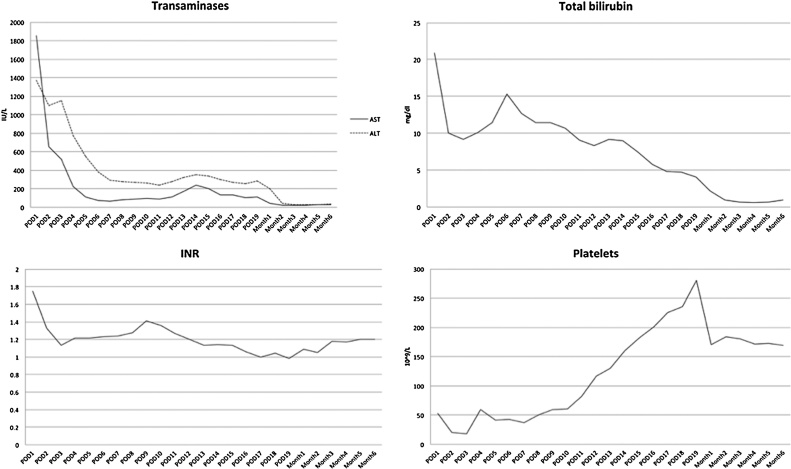
Postoperative trend of transaminases, bilirubin, INR and platelets.

Postoperative course was characterized by immediate graft function and no surgical complications. Due to low platelet count, anticoagulant treatment with enoxaparin 6000 IU was started on the 7th postoperative day and replaced by acetylsalicylic acid 200 mg o.d. one month after LT. Repeated Doppler ultrasound examinations and a CT scan performed in the postoperative period demonstrated patency of the reconstructed venous axis and otherwise regular graft perfusion (Fig. 1B). Patient made a good recovery and was discharged home on 19th postoperative day. At 6-month follow-up he is in good health, with normal hepatic function and regular graft perfusion (Fig. 4).

## 3 Discussion

Once considered a contraindication to LT, complex PVT still represents a technical challenge during LT and its incidence is likely underestimated in LT series, in which patients who are refused *a priori* based on PVT extent are not taken into account. Since the very early experiences with PVT management, it appeared that surgical technique has to be tailored to the anatomy of PVT, including its extension below the splenomesenteric confluence, availability of large portal vein collaterals and presence of spontaneous or surgical portosystemic shunts [7]. Whereas grade 1–2 PVT cases are most easily managed by low dissection of the portal vein towards the splenomesenteric confluence and thrombectomy [2,9], grade 4 PVT cases are more likely to require non-physiological reconstructions, i.e. those in which portal flow is not drained into the graft and after which portal hypertension persists.

At our Institution, which is approaching 3500 LT performed at a single center, all candidates to LT are screened for PVT by a contrast-enhanced computed tomography with 3D reconstructions. In last 10 years, PVT was diagnosed in 126 candidates to LT, classified as grade 1, 2 or 3 in 58 (46%), 46 (36.5%) and 22 (17.5%), respectively. In case grade 1–2 PVT is detected, low molecular weight heparin is started and, in patients who are suitable for, early TIPS is considered to preserve or reestablish portal vein patency [10,11].

In grade 3 PVT anticoagulants are more frequently ineffective and TIPS may be unfeasible due to the impossibility to identify a safe “landing point” in the portal venous system. Form a surgical standpoint, several options are available, of which mesoportal anastomosis using an interposition jump graft has been most frequently described.

In standard technique, the mesoportal interposition jump graft is placed in a transmesocolic, prepancreatic and retrogastric position [2,[5], [6], [7]]. The retrogastric passage is normally required to maintain the reconstructed venous axis straight, avoiding its kinking over pylorus and D1. Albeit the retrogastric passage may be omitted in particular cases, this requires the availability of a vascular patch of appropriate length, possibly obtained by using a double elongation patch. Surgical technique, however, has to be tailored to patient anatomy and surgical scenario.

In our case, we were forced to recur to a mesoportal interposition graft by the impossibility of using native portal vein due to its severe thickening and inflammatory changes. The jump graft was manufactured by using the iliac bifurcation and the proximal tract of common iliac veins, obtaining a graft of appropriate length and shape to get along the pancreatic head and D1, while avoiding any D1 or pylorus dissection or the need for a double elongation patch. In our practice, iliac vessels, if suitable, are always procured to allow possible vascular reconstructions in the recipient. In case pancreas is retrieved for transplantation, iliac vessels are shared with the pancreas team, according to each team necessities.

As feasibility of this technique depends upon the availability of IVC bifurcation, it could not be feasible when this has to be shared with other transplant teams, or in living donor LT. Furthermore, appropriate management of complex PVT is driven by careful evaluation of vascular anatomy and of the degree of portal hypertension. Thus, no “one-fits-all” approach can be recommended and our technique may not be appropriate in all grade 3 PVT cases.

## 4 Conclusion

In conclusion, we describe the “U-shaped” mesoportal jump graft, a technical variant of mesoportal jump graft to be employed in case of grade 3 PVT, when use of native portal vein is not possible. By this easy and reproducible technique, a graft of good length and appropriate shape can be obtained, possibly avoiding the need for its placement in a retrogastric position. Although long-term follow-up of our case is reassuring, further experience is necessary to confirm the value of the proposed technique in similar cases.

## Funding

Authors declare they received no funding for this paper.

## Ethical approval

The study is exempt from ethical approval at our Institution.

## Consent

The patient has given informed consent to the processing of personal data, including consent to the use of health data and images for scientific purposes.

## Registration of research studies

No registration was required for this study.

## Guarantor

Dott. Damiano Patrono.

## Provenance and peer review

Not commissioned, externally peer-reviewed.

## CRediT authorship contribution statement

**Damiano Patrono:** Conceptualization, Data curation, Writing - review & editing. **Sara Salomone:** Writing - original draft. **Carla Guarnaccia:** Data curation, Visualization. **Francesco Tandoi:** Writing - review & editing. **Francesco Lupo:** Writing - review & editing. **Paolo Fonio:** Writing - review & editing, Supervision. **Renato Romagnoli:** Writing - review & editing, Supervision.

## Declaration of Competing Interest

Authors declare they have no conflict of interest.
